# Supplementary material for: Integrating electric field modeling and neuroimaging to explain inter-individual variability of tACS effects
Source: Nat Commun. 2019 Nov 28;10:5427. doi: 10.1038/s41467-019-13417-6 (PMC6882891; doi:10.1038/s41467-019-13417-6)
Supplement: Supplementary file 1 — Supplementary Information [file 41467_2019_13417_MOESM1_ESM.pdf]

Supplementary Information

Integrating electric field modeling and neuroimaging to explain inter-individual variability of tACS effects

Kasten et al. Nature Communications. 2019

Supplementary Notes

Supplementary Note 1 – Predicting the maximum power within group specific cluster

Using a group ROI can introduce a bias such that larger power values are observed for participants whose α-power distribution on the source level is more similar to the cluster. We thus repeated our analysis identifying and averaging over the 1000 source locations within the clusters that show the strongest α-power increase to baseline for each participant. We submitted these power values to our linear regression analysis with factors CONDITION, $PRECISION_{Freq}$, $PRECISION_{Spat}$, and STRENGHT. We obtained similar results as for our ROI analysis in section 2.3. The model significantly predicted participants peak power increase in the ROI (multiple linear model, R^2 = .78, $F_{15,24}$ = 5.89, p < .001). Specifically, the factors CONDITION (same multiple linear model, β = 7.203e-25, t_{24} = 3.33, p = .003), the interaction between CONDITION, $PRECISION_{Freq}$ and STRENGTH (same multiple linear model, β = 5.114e-23, t_{24} = 3.24, p = .003) and the interaction between CONDITION, $PRECISION_{Freq,}$ $PRECISION_{Spat}$ and STRENGTH (same multiple linear model, β = 2.896-22, t_{24} = 4.07, p < .001) significantly predicted the power increase. When separately fitted to the data of the two groups, the model again failed to explain the power increase in the sham group (multiple linear model, R^2 = .15, $F_{7,12}$ = 0.31, p = .93), but significantly predicts the power increase after tACS (multiple linear model, R^2 = .82, $F_{7,12}$ = 7.58, p = .001). Specifically, the factors $PRECISION_{Spat}$ (same multiple linear model, β = 3.90-24, t_{12} = 3.74, p = .003), the interactions between $PRECISION_{Spat}$ and $PRECISION_{Freq}$ (same multiple linear model, β = 4.41-24, t_{12} = 2.73, p = .018), STRENGTH and $PRECISION_{Freq}$ (same multiple linear model, β = 4.53-23, t_{12} = 4.89, p < .001) and $PRECISION_{Freq}$, $PRECISION_{Spat}$ and STRENGTH (same multiple linear model, β = 2.90-22, t_{12} = 6.56, p < .001) significantly predicted participants peak power increase after tACS.

Supplementary Note 2 – Outlier analysis Experiment 1

Inspection of the data of experiment 1 may suggest the presence of extreme values/outliers in the data (Fig. 3b). Such extreme values can artificially drive the observed relationship between our predictors and the power increase after stimulation. Z-transformation of the power increase after stimulation within the group specific ROIs revealed two values exceeding two standard deviations from the mean of the whole sample ($Z_{outlier1}$ = 2.49, $Z_{outlier2}$ = 4.34). In order to test whether these two datapoints excessively drove the model fits, we repeated the regression analysis applied to the tACS group after removing the two datapoints. Without the two datapoints, the model still accounts for 62% of the variance (multiple linear model, R^2 = .62, $F_{7,10}$ = 2.34, p = .11), although not reaching significance. The factor $\text{PRECISION}_{\text{Spat}}$ (same multiple linear model, β = 1.30e-24, t_{10} = 3.16, p = .01) significantly predicted the data. Further, the model showed trends for the $\text{STRENGTH*PRECISION}_{\text{Freq}}$ (same multiple linear model, β = 1.25e-23, t_{10} = 2.20, p = .052) and the $\text{STRENGTH*PRECISION}_{\text{Freq}}\text{*PRECISION}_{\text{Spat}}$ interactions (same multiple linear model, β = 8.77e-23, t_{10} = 1.79, p = .10). While the model performs weaker after removal of the two datapoints (which can at least in part be explained by reduced sensitivity of the model due to the smaller sample size), it still generally supports the direction of our findings. In a next step, we tested whether this model trained on the remaining datapoints would predict the occurrence of these outliers based on their electric field parameters and stimulation frequency mismatches. As depicted in Supplementary Figure 3, the model tends to underestimate the power increase after stimulation in these two datapoints. However, remarkably, the model accurately predicts both datapoints to be extreme values as compared to the rest of the sample. Taken together, we conclude that while the two datapoints reflect extreme values as compared to the rest of the sample, they are in agreement with the model predictions. I.e. compared to the rest of the sample, the spatial extend of the electric field, its intensity and the stimulation frequency were most optimal and thus gave rise to the strong power increase that was observed after stimulation.

Supplementary Note 3 – Debriefing Experiment 1

Overall 16 out of 40 participants indicated that they believed to have received electrical stimulation during the experiment (11 out of 20 in the sham group and 5 out of 20 in the tACS group). A Pearson's Chi-Squared test for count data revealed no significant difference for the number of 'yes' and 'no' answers between groups (X_1^2 = 2.6, p = .11). In addition, we asked participants to rate their confidence in the answer on a scale from 1 to 10. Participants' ratings were submitted to a 2x2 factorial Analysis of Variance (ANOVA) with between subject factors CONDITION (tACS vs. sham) and ANSWER (yes vs. no). The ANOVA revealed neither an effect of CONDITION ($F_{1,36}$ = 2.00, p = .16, η^2 = 0.05) or ANSWER ($F_{1,36}$ = 0.007, p = .92, η^2 < 0.01), nor an interaction ($F_{1,36}$ = 0.04, p = .54, η^2 = 0.01). On average participant's confidence was in the upper-medium range of the rating scale (tACS+yes: 7 ± 0.7, tACS+no: 6.33 ± 2.58, sham+yes: 5.18 ± 2.89, sham+no: 5.56 ± 2.31).

Supplementary Note 4 – Debriefing Experiment 2

One participant did not completely fill out the debriefing questionnaire and had to be excluded from the debriefing analysis. Of the remaining 18 participants 11 correctly indicated that they thought they had received tACS after the tACS session, while 7 indicated to believe not to have received active stimulation. After sham stimulation 12 participants indicated they thought to have received tACS, while 6 indicated that they did not thought to have received stimulation. A Pearson's Chi-Squared test for count data revealed no significant difference for the number of 'yes' and 'no' answers between experimental sessions (X_1^2= 0, p = 1). There was no effect of CONDITION ($F_{1,32}$ = 2.54, p = .12, η^2 = 0.07), or ANSWER ($F_{1,32}$ = 0.39, p = .53, η^2 = 0.01) and no interaction effect ($F_{1,32}$ = 0.04, p = .85, η^2 < 0.01) on participants' confidence ratings. On average participants' confidence was in the upper medium range (tACS+yes: 5.36 ± 2.2, tACS+no: 6 ± 2.2, sham+yes: 6.7 ± 2.1, sham+no: 7 ± 2.6).

Overall, results of the debriefing indicate that participants in both experiments were successfully blinded towards their experimental condition.

Supplementary Figures

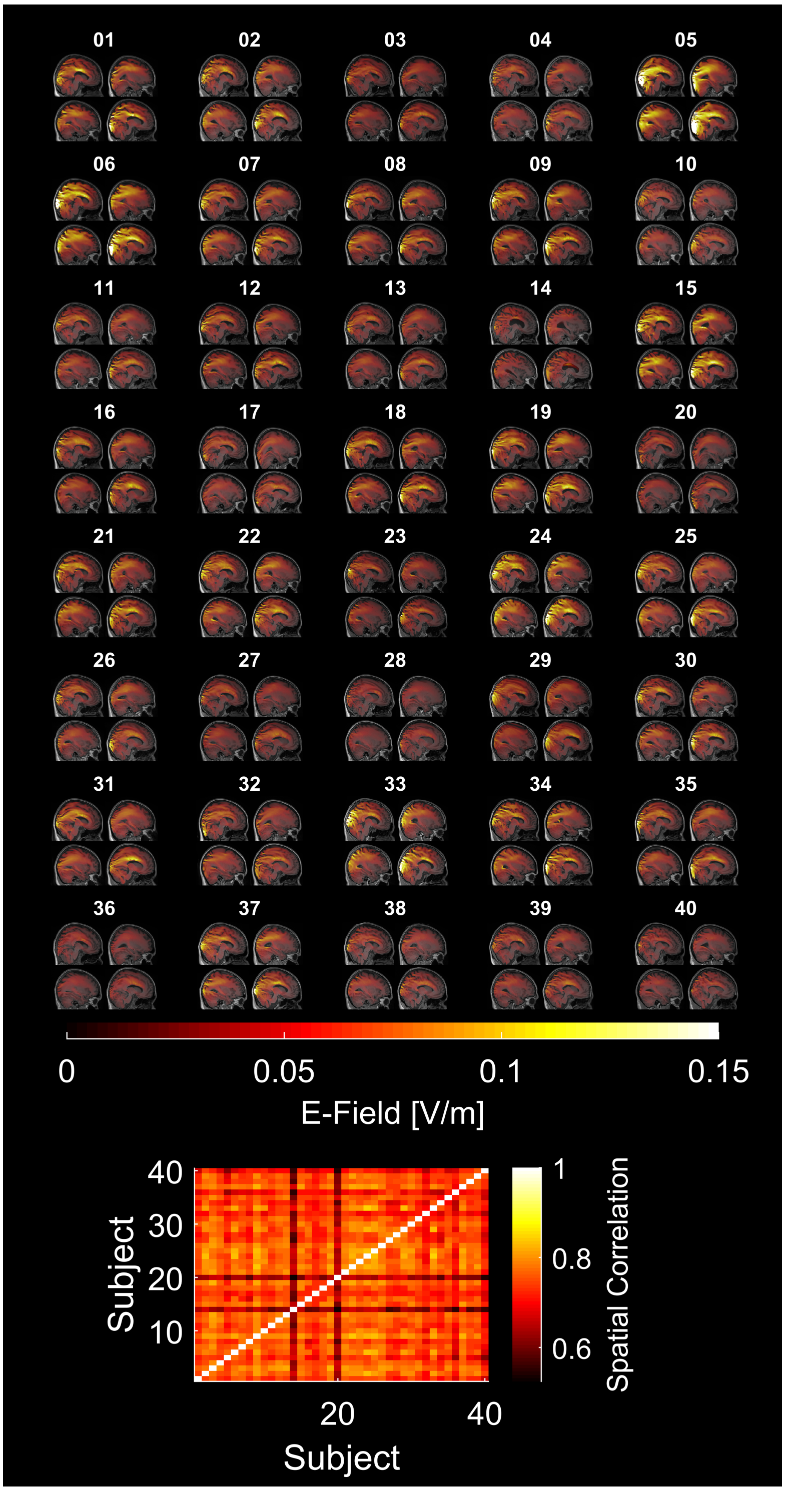

Supplementary Figure 1: Variability of electric fields across all subjects. (Top) Simulations of the electric fields inside the brain resulting from the Cz-Oz configuration applied at 1 mA (peak-to-peak) shown for all subjects. Simulations were performed on the individual brain and warped into MNI space for visualization purposes. Overall simulation results show a quite large variability between subjects. Please refer to Supplementary Fig. S1 for an overview of all simulations in the sample (**Bottom**) Spatial correlations of electric fields between all subjects in MNI-space.

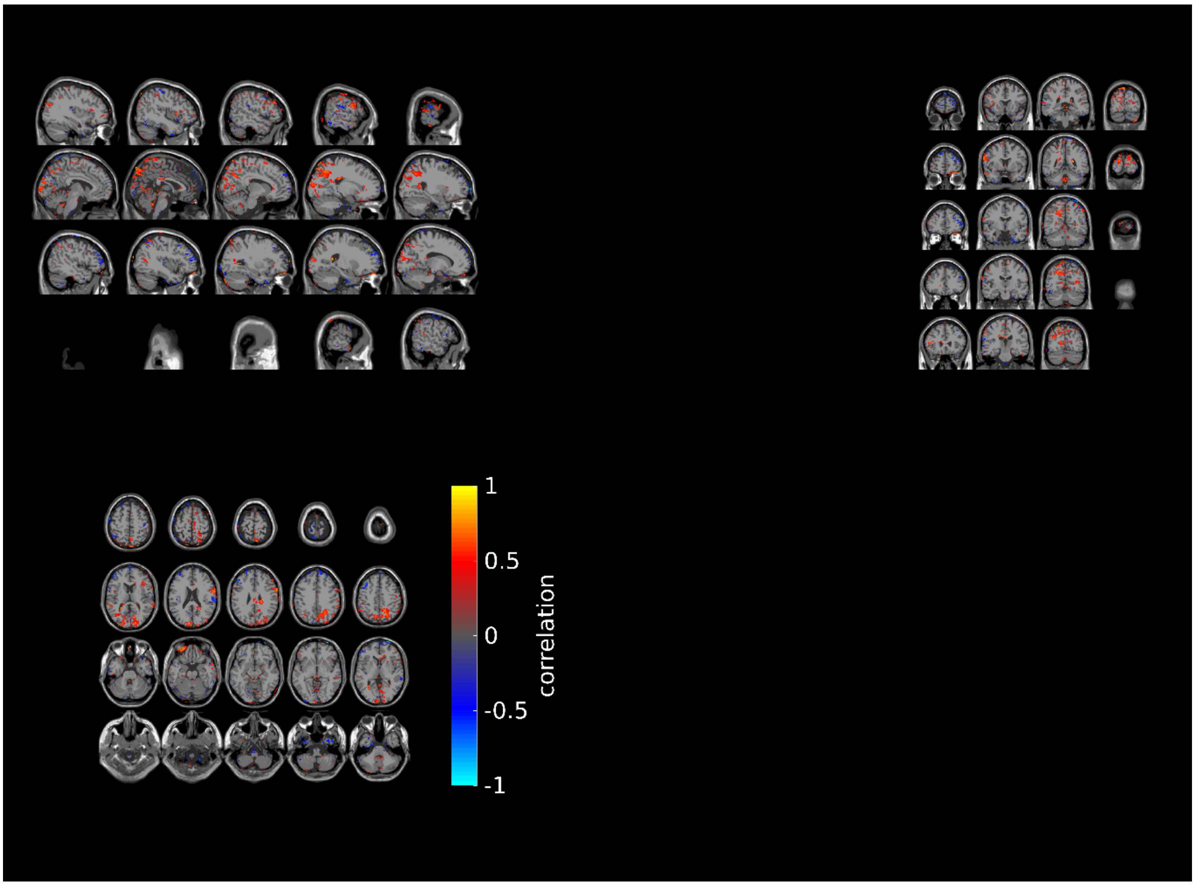

Supplementary Figure 2: Voxel-wise correlation between electric field magnitude and tACS effect. Correlation between the simulated electric field and the individually sham controlled tACS effect in the α-band (α-power increase relative to baseline after tACS – α-power increase relative to baseline after sham). Pearson's correlation coefficient between the simulated electric field warped into MNI space and the tACS effect was computed across subjects for each voxel. The resulting correlation maps are thresholded at a significance level of $p < .05$ (uncorrected).

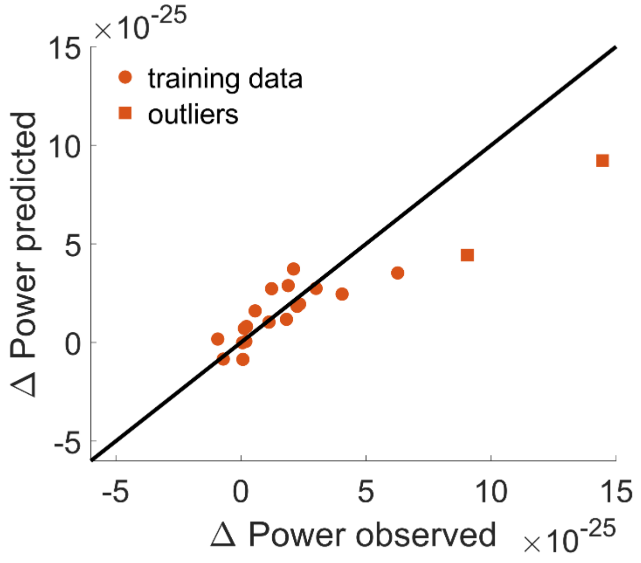

Supplementary Figure 3: Linear model predicts outliers in experiment 1. Scatterplots depict the power increase from the baseline to the post-stimulation block predicted by the statistical model trained on the tACS group after removing two extreme values. Predictions of the model are plotted against empirically observed values. Each dot represents data of a single subject. Squares indicate the power increase for the two extreme values predicted by the model, plotted against the observed power increase. Although the power increase of the extreme values is underestimated. The model accurately predicts both extreme values to be lager than the rest of the sample. The diagonal indicates the line of perfect prediction (predicted Δpower = observed Δpower). Source data are provided as a Source Data file.

Supplementary Tables

Model	AIC
CONDITION*PRECISION$_{Spat}$*PRECISION$_{Freq}$*STRENGTH (final model)	-4429.8
CONDITION* PRECISION$_{Spat}$*STRENGTH	-4404.8
CONDITION* PRECISION$_{Spat}$* PRECISION$_{Freq}$	-4405.2
CONDITION* PRECISION$_{Freq}$*STRENGTH	-4403.5
CONDITION* STRENGTH	-4403.1
CONDITION* PRECISION$_{Freq}$	-4399.7
CONDITION* PRECISION$_{Spat}$	-4412.1
CONDITION	-4403.2
PRECISION$_{Spat}$*PRECISION$_{Freq}$*STRENGTH	-4405.1
PRECISION$_{Spat}$* STRENGTH	-4405.1
PRECISION$_{Spat}$*PRECISION$_{Freq}$	-4402.7
PRECISION$_{Freq}$* STRENGTH	-4405.2
STRENGTH	-4401.2
PRECISION$_{Freq}$	-4400.9
PRECISION$_{Spat}$	-4405.7
Intercept	-4402.8

Supplementary Table 1: Comparison of Akaike's Information Criterion (AIC) across all possible models for the first regression analysis. AIC clearly favors the full model (top).

Model	AIC
$PRECISION_{Spat}$*$PRECISION_{Freq}$*STRENGTH (final model)	-2217.3
$PRECISION_{Spat}$* STRENGTH	-2192.3
$PRECISION_{Spat}$*$PRECISION_{Freq}$	-2192.6
$PRECISION_{Freq}$* STRENGTH	-2192.1
STRENGTH	-2190.9
$PRECISION_{Freq}$	-2188.8
$PRECISION_{Spat}$	-2196.1
Intercept	-2190.5

Supplementary Table 2: Comparison of Akaike's Information Criterion (AIC) across all possible models for the second regression analysis fitted to data of the tACS group. Again, AIC clearly favors the full model (top).

Model	AIC
$PRECISION_{Spat}$*$PRECISION_{Freq}$*STRENGTH (final model)	-2211.0
$PRECISION_{Spat}$* STRENGTH	-2218.5
$PRECISION_{Spat}$*$PRECISION_{Freq}$	-2218.4
$PRECISION_{Freq}$* STRENGTH	-2216.5
STRENGTH	-2220.2
$PRECISION_{Freq}$	-2220.2
$PRECISION_{Spat}$	-2221.5
Intercept	-2222.1

Supplementary Table 3: Comparison of Akaike's Information Criterion (AIC) across all possible models for the second regression analysis fitted to data of the sham group. Here, AIC favors the intercept model omitting all other predictors. To allow direct comparisons of the model fits between the groups, the full model (top) was fitted to the data of the sham group.

Model	AIC
$PRECISION_{Freq}$*$STRENGTH_{Eye}$* $STRENGTH_{Skin}$ (full model)	-2181.5
$PRECISION_{Freq}$*$STRENGTH_{Eye}$	-2186.7
$PRECISION_{Freq}$*$STRENGTH_{Skin}$	-2185.5
$STRENGTH_{Eye}$* $STRENGTH_{Skin}$	-2187.8
$STRENGTH_{Eye}$	-2190.4
$STRENGTH_{Skin}$	-2189.0
$PRECISION_{Freq}$	-2188.8
Intercept (best model)	-2190.5

Supplementary Table 4: Comparison of Akaike's Information Criterion (AIC) across all possible models for the alternative model incorporating the strength of peripheral stimulation of the skin and the eyeballs. The model was fitted to data of the tACS group. AIC favors the intercept model omitting all other predictors.

ID	IAF before experiment	tACS frequency	IAF during baseline	Mismatch tACS/baseline
01	11.5	11	11.25	-0.25
02	10	10	10	0
03	11	11	11	0
04	11	11	11.5	-0.5
05	10	10	11.5	-1.5
06	11	11	11.25	-0.25
07	10	10	10.5	-0.5
08	8	8	11.25	-3.25
09	9.5	10	11.5	-1.5
10	9.5	10	10.25	-0.25
11	10.5	10	8.5	1.5
12	10.5	10	10.5	-0.5
13	10	10	9.5	0.5
14	12	12	11.5	0.5
15	10	10	10	0
16	11	11	10.25	0.75
17	10	10	10.5	-0.5
18	8.5	8	8	0
19	10.5	11	10.5	0.5
20	11.5	12	11	1
21	11	9	11	-2
22	9.5	10	9.5	0.5
23	11	11	11	0
24	10	10	10	0
25	9	9	8.75	0.25
26	9.5	9	9.25	-0.25
27	9	9	8	1
28	9	9	9.25	-0.25
29	10	10	10	0
30	9.5	9	8.25	0.75
31	11	11	10.5	0.5
32	11.5	11	8	3
33	10	10	9.75	0.25
34	8	8	8	0
35	11	11	11.25	-0.25
36	11	11	11.25	-0.25
37	10.5	10	10.5	-0.5
38	10	10	10	0
39	10.5	11	10	0.5

40	10.5	11	10.75	0.25

Supplementary Table 5: Overview of individual α-frequency measured before the experiment, stimulation frequency during the experiment, IAF measured during the baseline block and the mismatch between the tACS frequency and the IAF during the baseline block of the first experiment.

ID	IAF before experiment	tACS frequency	IAF during baseline	Mismatch tACS/baseline
01a	10.5	11	10	1
01b	10.5	11	10	1
02a	9.5	9	9	0
02b	9.5	9	9.25	-0.25
03a	8.5	8	8.5	-0.5
03b	9	9	7.75	1.25
04a	11	11	10	1
04b	11	11	9.5	1.5
05a	9.5	10	9.75	0.25
05b	9.5	9	9.75	-0.75
06a	9.5	9	8.5	0.5
06b	8.5	9	10	-1
07a	11.5	12	11.25	0.75
07b	11.5	12	11.25	0.75
08a	10.5	11	10.75	0.25
08b	10.5	11	10.5	0.5
09a	12	12	11	1
09b	12	12	11	1
10a	9.5	10	8.5	1.5
10b	10	10	9.75	0.25
11a	12	12	11.75	0.25
11b	11.5	12	11.25	0.75
12a	11.5	12	10.25	1.75
12b	11.5	11	9	2
13a	11	11	10.75	0.25
13b	11	11	11	0
14a	10	10	10	0
14b	10	10	10-25	-0.25
15a	9.5	10	9.5	0.5
15b	9.5	9	9.5	-0.5
16a	10.5	11	10.75	0.25
16b	10	10	12.25	-2.25
17a	9.5	10	10.75	-0.75
17b	11	11	10.75	0.25
18a	11	11	11.25	-0.25
18b	10.5	11	11.25	-0.25
19a	10.5	11	10.75	0.25
19b	10.5	10	10.5	-0.5

Supplementary Table 6: Overview of individual α-frequency measured before the experiment, stimulation frequency during the experiment, IAF measured during the baseline block and the mismatch between the tACS frequency and the IAF during the baseline block of the second experiment.

Supplementary Methods

Participants' individual α-frequency was determined by averaging power spectra over the following planar gradiometer channels:

'MEG1632', 'MEG1633', 'MEG1732', 'MEG1733', 'MEG1742', 'MEG1743', 'MEG1842', 'MEG1843', 'MEG1912', 'MEG1913', 'MEG1922', 'MEG1923', 'MEG1932', 'MEG1933', 'MEG1942', 'MEG1943', 'MEG2012', 'MEG2013', 'MEG2022', 'MEG2023', 'MEG2032', 'MEG2033', 'MEG2042', 'MEG2043', 'MEG2112', 'MEG2113', 'MEG2122', 'MEG2123', 'MEG2132', 'MEG2133', 'MEG2232', 'MEG2233', 'MEG2312', 'MEG2313', 'MEG2322', 'MEG2323', 'MEG2332', 'MEG2333', 'MEG2342', 'MEG2343', 'MEG2432', 'MEG2433', 'MEG2442', 'MEG2443', 'MEG2512', 'MEG2513', 'MEG2542', 'MEG2543'
