# Supplementary material for: Integrating electric field modeling and neuroimaging to explain inter-individual variability of tACS effects
Source: Nat Commun. 2019 Nov 28;10:5427. doi: 10.1038/s41467-019-13417-6 (PMC6882891; doi:10.1038/s41467-019-13417-6)
Supplement: Supplementary file 3 — Reporting Summary [file 41467_2019_13417_MOESM3_ESM.pdf]

nature research

Corresponding author(s): Prof. Dr. Christoph S. Herrmann

Last updated by author(s): Oct 23, 2019

Reporting Summary

Nature Research wishes to improve the reproducibility of the work that we publish. This form provides structure for consistency and transparency in reporting. For further information on Nature Research policies, see Authors & Referees and the Editorial Policy Checklist.

Statistics

For all statistical analyses, confirm that the following items are present in the figure legend, table legend, main text, or Methods section.

n/a	Confirmed	
☐	☒	The exact sample size (n) for each experimental group/condition, given as a discrete number and unit of measurement
☐	☒	A statement on whether measurements were taken from distinct samples or whether the same sample was measured repeatedly
☐	☒	The statistical test(s) used AND whether they are one- or two-sided<br>*Only common tests should be described solely by name; describe more complex techniques in the Methods section.*
☒	☐	A description of all covariates tested
☐	☒	A description of any assumptions or corrections, such as tests of normality and adjustment for multiple comparisons
☐	☒	A full description of the statistical parameters including central tendency (e.g. means) or other basic estimates (e.g. regression coefficient) AND variation (e.g. standard deviation) or associated estimates of uncertainty (e.g. confidence intervals)
☐	☒	For null hypothesis testing, the test statistic (e.g. F, t, r) with confidence intervals, effect sizes, degrees of freedom and P value noted<br>*Give P values as exact values whenever suitable.*
☒	☐	For Bayesian analysis, information on the choice of priors and Markov chain Monte Carlo settings
☒	☐	For hierarchical and complex designs, identification of the appropriate level for tests and full reporting of outcomes
☐	☒	Estimates of effect sizes (e.g. Cohen's d, Pearson's r), indicating how they were calculated

Our web collection on statistics for biologists contains articles on many of the points above.

Software and code

Policy information about availability of computer code

Data collection	MEG and MRI data acquisition was performed using the corresponding data acquisition Software of the manufacturers (MEG: Elekta Neuromag Triux System, Elekta Oy, Helsinki, Finnland; MRI: Siemens Magnetom Prisma 3T)
Data analysis	Data analysis was performed in Matlab 2016a (The MathWorks, Inc. Natick, MA, USA) using the Fieldtrip toolbox (Oostenfeld et al. 2011) for MEG data processing and ROAST v. 2.7 (Huang et al, 2017) for individualized electric field modeling.

For manuscripts utilizing custom algorithms or software that are central to the research but not yet described in published literature, software must be made available to editors/reviewers. We strongly encourage code deposition in a community repository (e.g. GitHub). See the Nature Research guidelines for submitting code & software for further information.

Data

Policy information about availability of data

All manuscripts must include a data availability statement. This statement should provide the following information, where applicable:

- Accession codes, unique identifiers, or web links for publicly available datasets
- A list of figures that have associated raw data
- A description of any restrictions on data availability

The data that support the findings of this study are available upon reasonable request from the corresponding author CSH. The data are not publicly available due to potentially identifying information that could compromise participant privacy.

Field-specific reporting

Please select the one below that is the best fit for your research. If you are not sure, read the appropriate sections before making your selection.

☒ Life sciences ☐ Behavioural & social sciences ☐ Ecological, evolutionary & environmental sciences

For a reference copy of the document with all sections, see nature.com/documents/nr-reporting-summary-flat.pdf

Life sciences study design

All studies must disclose on these points even when the disclosure is negative.

Sample size	An a priori power analysis was performed to determine the sample size. Based on previous studies the effect of tACS on alpha power is usually relatively large (eta^2 > 0.2, cohen's d > 1). Based on these effects, a sample size of at least 36 participants is required to achieve a statistical power (1-beta = 0.9). We thus decided for a sample size of 40 participants in study I (between subject design) and 20 participants in study II (within subject design.
Data exclusions	No data was excluded in study I. In study II one subject aborted the experiment, two indicated to not have slept the night before both measurement sessions. As high tiredness is problematic with respect to the induction of plasticity (which is the assumed mechanism behind tACS aftereffects) and the recording of alpha oscillations we excluded these subjects from the analysis.
Replication	Leave-one-out cross validation was performed to test whether the statistical model can predict new data well. Results suggest that this is the case. The experiment was then repeated in a second sample using a within-subject design.
Randomization	Randomization was carried out by MATLAB code. A constraint was implemented to ensure equal numbers of male and female participants across experimental groups.
Blinding	Assignments to experimental groups were carried out by MATLAB code. However, due to a large artifact in MEG signals during stimulation, the experimental condition cannot be disguised to the experimenters.

Reporting for specific materials, systems and methods

We require information from authors about some types of materials, experimental systems and methods used in many studies. Here, indicate whether each material, system or method listed is relevant to your study. If you are not sure if a list item applies to your research, read the appropriate section before selecting a response.

Materials & experimental systems

n/a	Involved in the study	
☒	☐	Antibodies
☒	☐	Eukaryotic cell lines
☒	☐	Palaeontology
☒	☐	Animals and other organisms
☐	☒	Human research participants
☒	☐	Clinical data

Methods

n/a	Involved in the study	
☒	☐	ChIP-seq
☒	☐	Flow cytometry
☒	☐	MRI-based neuroimaging

Human research participants

Policy information about studies involving human research participants

Population characteristics	40 healthy volunteers (age: 24 ± 3 years, 20 females, 20 males) without history of neurological or psychiatric disease participated in study I. 19 healthy volunteers (age: 25 ± 3 years, 11 females) without history of neurological or psychiatric disease participated in study II.
Recruitment	Participants were recruited via the digital black-board of the University of Oldenburg
Ethics oversight	Commission for Research Impact Assessment and Ethics at the University of Oldenburg

Note that full information on the approval of the study protocol must also be provided in the manuscript.
